# Pristine and Hydroxylated Fullerenes Prevent the Aggregation of Human Islet Amyloid Polypeptide and Display Different Inhibitory Mechanisms

**DOI:** 10.3389/fchem.2020.00051

**Published:** 2020-02-05

**Authors:** Cuiqin Bai, Zenghui Lao, Yujie Chen, Yiming Tang, Guanghong Wei

**Affiliations:** State Key Laboratory of Surface Physics, and Key Laboratory for Computational Physical Science (Ministry of Education), Department of Physics, Multiscale Research Institute of Complex Systems, Fudan University, Shanghai, China

**Keywords:** type 2 diabetes, hIAPP aggregation, inhibitory mechanism, replica exchange molecular dynamics simulations, C_60_

## Abstract

Protein aggregation, involving the formation of dimers, oligomers, and fibrils, is associated with many human diseases. Type 2 diabetes is one of the common amyloidosis and linked with the aggregation of human islet amyloid polypeptide (hIAPP). A series of nanoparticles are reported to be able to interact with proteins and enhance/inhibit protein aggregation. However, the effects of C_60_ (a model system of hydrophobic nanoparticle) and C_60_(OH)_8_ (a hydroxylated fullerene) on hIAPP aggregation remain unknown. In this study, we investigate the influences of pristine fullerene C_60_ and hydroxylated C_60_ on the dimerization of hIAPP using molecular dynamics (MD) simulations. Extensive replica exchange molecular dynamics (REMD) simulations show that isolated hIAPP dimers adopt β-sheet structure containing the amyloid-precursor (β-hairpin). Both C_60_ and C_60_(OH)_8_ notably inhibit the β-sheet formation of hIAPP dimer and induce the formation of collapsed disordered coil-rich conformations. Protein—nanoparticle interaction analyses reveal that the inhibition of hIAPP aggregation by C_60_ is mainly via hydrophobic and aromatic-stacking interactions, while the prevention of hIAPP aggregation by C_60_(OH)_8_ is mostly through collective hydrogen bonding and aromatic-stacking interactions. Conventional MD simulations indicate that both C_60_ and C_60_(OH)_8_ weaken the interactions within hIAPP protofibril and disrupt the β-sheet structure. These results provide mechanistic insights into the possible inhibitory mechanism of C_60_ and C_60_(OH)_8_ toward hIAPP aggregation, and they are of great reference value for the screening of potent amyloid inhibitors.

## Introduction

Human islet amyloid polypeptide **(**hIAPP) is an intrinsically disordered protein and plays a significant role in the progression of type 2 diabetes (Cooper et al., 1987). hIAPP has a high propensity to form amyloid aggregates (Larson and Miranker, 2004; Brender et al., 2010). Amyloid deposits derived from hIAPP are observed in human islet extracellular space in type 2 diabetes and the formation of intracellular hIAPP oligomers may conduce to β-cell loss in Type 2 diabetes (Haataja et al., 2008). Inhibition of hIAPP aggregation and destabilization of preformed hIAPP fibrils are considered as two major therapeutic strategies for treating Type 2 diabetes. Finding an effective inhibitor of hIAPP aggregation is a crucial step for reducing islet β-cells death and the development of drugs against Type 2 diabetes. Researchers have made great efforts to search for inhibitors against hIAPP aggregation. Increasing experimental studies show that peptides (Yan et al., 2006; Abedini et al., 2007; Saunders et al., 2016), and natural small molecules (Cao and Raleigh, 2012; Palhano et al., 2013; Young et al., 2015; Pithadia et al., 2016) can modulate hIAPP aggregation and drive the peptides into disordered off-pathway aggregates which almost has no toxicity. Very recently, Ke et al. demonstrated that nanomaterials can inhibit hIAPP aggregation and reduce the toxicity *in silico, in vitro*, and *in vivo* (Wang et al., 2018; Faridi et al., 2019; Ke et al., 2019).

Carbon nanoparticles including graphene, carbon nanotube, fullerene, and its derivatives (especially hydroxylated fullerenes) have also been of great concern due to their excellent physicochemical properties (Mahmoudi et al., 2013) [such as high capacity to cross biological barriers (Tsuchiya et al., 1996; Sumner et al., 2010), low biotoxicity (Zhu et al., 2007), and high solubility (Da Ros and Prato, 1999; Maciel et al., 2011)]. Experimental studies have demonstrated that fullerenes and their derivatives can prevent the aggregation of amyloid proteins. For example, pristine fullerenes, carboxyfullerenes, and hydroxylated fullerene, strongly inhibit the aggregation of Aβ and Aβ fragments (Dugan et al., 1997; Kim and Lee, 2003; Podolski et al., 2007; Bobylev et al., 2011). Hydroxylated carbon nanotubes can significantly impede the aggregation of hIAPP (Mo et al., 2018). Graphene quantum dots are able to prevent the aggregation of hIAPP and reduce the toxicity *in vivo* (Wang et al., 2018). On the computational side, researchers investigated the interactions of amyloid proteins and carbon nanoparticles at atomic level of details with an attempt to uncover the underlying inhibitory mechanisms. By atomistic replica exchange molecular dynamics (REMD) simulations, Li et al. found that carbon nanotube can significantly suppress the formation of β-sheet rich Aβ_16−22_ oligomers (Li et al., 2011). Using the same simulation method, Xie et al. explored the effect of different size of fullerenes on the aggregation of Aβ_16−22_. Their simulations showed that fullerene C_180_, albeit with a smaller surface area than 3C_60_, exhibits an unexpectedly more effective inhibition of β-sheet formation. The stronger inhibition of β-sheet formation by C_180_ is due to the stronger hydrophobic and aromatic-stacking interactions between the fullerene hexagonal rings and the Phe rings than that between the pentagonal rings and the Phe rings (Xie et al., 2014). MD simulations revealed that C_60_ can destabilize Aβ protofibrils by disrupting the D23–K28 salt bridge (Andujar et al., 2012; Zhou et al., 2014). Guo et al. explored the influences of graphene, carbon nanotube, and C_60_ on oligomerization of IAPP_22−28_ fragment and found that these carbon nanoparticles inhibit the formation of the β-sheet-rich oligomers (Guo et al., 2013). However, questions remain to be addressed. For example, can pristine C_60_ inhibit the aggregation of full length hIAPP and disrupt hIAPP protofibrils? If yes, what is the inhibitory mechanism and how different is it from that of hydroxylated C_60_?

In this work, we conducted extensive explicit solvent replica-exchange molecular dynamics (REMD) simulations on hIAPP dimer with and without four C_60_/C_60_(OH)_8_ nanoparticles. Our aim is to explore the effects of pristine and hydroxylated C_60_ nanoparticles on full-length hIAPP aggregation. REMD simulations showed that both C_60_ and C_60_(OH)_8_ display a strong inhibition of β-sheet formation. The nanoparticle—peptide interactions analyses revealed that the strong β-sheet inhibition results from the strong binding of C_60_/C_60_(OH)_8_ to hIAPP. C_60_ preferentially binds to the hydrophobic residues and aromatic residues, while C_60_(OH)_8_ has a relatively high probability to bind to hydrophilic residues and aromatic residues. In addition, to examine whether C_60_/C_60_(OH)_8_ nanoparticles can disrupt the preformed protofibril, we carried out conventional MD simulations for hIAPP protofibril in the absence and presence of C_60_/C_60_(OH)_8_. The MD simulations revealed that both C_60_ and C_60_(OH)_8_ can disrupt the β-sheet structure and destabilize hIAPP protofibril.

## Materials and Methods

### Systems

#### The hIAPP Dimer Systems

The hIAPP dimer with/without C_60_/C_60_(OH)_8_ nanoparticles, were simulated, and they were denoted as hIAPP-dimer, hIAPP-dimer + C_60_ and hIAPP-dimer + C_60_(OH)_8_. hIAPP has 37 amino acid residues (with sequence KCNTATCATQ^10^ RLANFLVHSS^20^ NNFGAILSST^30^ NVGSNTY) with an amidated C-terminus and a disulfide bond forming between Cys2 and Cys7. In accordance with previous experimental studies (Nanga et al., 2011), the N-terminus, the side chains of Lys1 and Arg11 were protonated (NH_3_^+^, Lys^+^, and Arg^+^). And the sidechain of H18 was uncharged to mimic the experimental conditions with pH of ~7.3 (Goldsbury et al., 2000). It is true that the protonation state of His will change along with the local environment changes. As done recently by other groups (Dupuis et al., 2009, 2011; Deng et al., 2013; Qiao et al., 2013), we neglected the pKa shift in all of our simulations as the involvement of pKa calculation in MD simulations makes it very computationally expensive. Every hIAPP dimer was put in the center of a cubic box with a side length of 6.7 nm. Four C_60_ or four C_60_(OH)_8_ molecules were displaced in the solvated peptide system at random thus nanoparticle: peptide is 2:1 at molar ratio, which were consistent with previous simulation studies (Bai et al., 2019). The partial charges of oxygen and hydrogen atoms in hydroxyl groups were −0.8 and +0.3, and that of carbon atoms bonded with hydroxyl groups in C_60_(OH)_8_ was +0.5, while other carbon atoms were uncharged (Goldsbury et al., 2000). Six counter ions (Cl^−^) were added to the three systems for neutralization. More details about the system preparation could be found in the Supporting Information section.

#### The hIAPP Protofibril Systems

The hIAPP protofibril with/without C_60_/C_60_(OH)_8_ nanoparticles, are simulated, and they are denoted as hIAPP-protofibril, hIAPP-protofibril + C_60_ and hIAPP-protofibril + C_60_(OH)_8_ systems. Kindly provided by Professor Tycko (Luca et al., 2007), the initial structure of hIAPP protofibril is a hIAPP decamer including two rotationally symmetric protofibrillar pentamers, Each pentamer contains two anti-parallel β-strand, β1 (spanning residues 8–17) and β2 (spanning residues 28–37), and a “loop” (spanning residues 18–27). The three regions together formed a U-shaped structure. There are 20 C_60_/C_60_(OH)_8_ nanoparticles in hIAPP-protofibril + C_60_/C_60_(OH)_8_ systems (nanoparticle: peptide = 2:1 in molar ratio). Thirty counter ions (Cl^−^) are added to the systems for neutralization. C_60_/C_60_(OH)_8_ nanoparticles are randomly distributed in the simulation box. The dimensions of the simulation box are 9.5 × 9.5 × 9.5 nm^3^ for all the three systems.

### REMD and MD Simulations

Both REMD and MD simulations are performed in the isothermal-isobaric (NPT) ensemble at a pressure of 1 bar using GROMACS−4.5.3 software package (Sugita and Okamoto, 1999; Nadler and Hansmann, 2008). We choose OPLS force field TIP4P water molecules, consistent with previous computational studies of hIAPP (Qi et al., 2014; Mo et al., 2016; Bai et al., 2019). hIAPP molecules are kept from the water box at least for 1.0 nm. There are 48 replicas for each system in the REMD simulations, at different temperatures exponentially distributed from 306 to 409 K. Every 1,000 integration steps, two adjacent replicas attempt to exchange with an average acceptance ratios of ~15% for each system. The integration time step is 2 fs. Each replica was simulated for 360 ns, and thus the accumulative simulation time period for each system was 17.28 μs. For MD simulations, we perform two individual 300 ns simulations for each of the three systems: hIAPP-protofibril and hIAPP-protofibril + C_60_ and hIAPP-protofibril + C_60_(OH)_8_.

### Analysis Methods

The tools implemented in GROMACS software package and our in-house developed codes were both used to analyze the trajectories. For REMD simulations, we chose the last 160 ns simulation data for analysis as the first 200 ns data of each replica may have bias of the initial structures. Daura method was used for cluster analysis with a C_α_-root-mean-square deviation (C_α_-RMSD) cutoff of 0.35 nm (Daura et al., 1999). We analyzed the REMD trajectories by calculating the secondary structure propensities by the DSSP program, number of hydrogen bonds (H-bonds), percentage of β-strand length, free energy landscape (or potential of mean force), hIAPP–nanoparticles binding probabilities, and hIAPP–nanoparticles contact surface area (CSA), pairwise residue contact probabilities of both main chain-main chain (MC-MC) contact and side chain-side chain (SC-SC) contact. For MD simulations, H-bond number within hIAPP protofibril and between hIAPP and nanoparticles, secondary structure probabilities of hIAPP and the hIAPP–nanoparticle binding probability were calculated. The VMD (Humphrey et al., 1996) and Pymol (Schrodinger, 2015) programs were used for graphical structure analysis and trajectory visualization.

## Results

For each system at 310 K, we examined the data convergence within two different time intervals (200–280 and 280–360 ns) before analyzing the REMD simulation data by comparing four parameters as followed. These parameters include the probability density functions (PDF) of the radius of gyration (Rg) and the hydrogen bond (H-bond), the probabilities of coil, β-sheet and helix structure of each amino acid residue. As it can be seen in Figures S2–S5, the simulation data from the two independent time periods coincide very well in terms of all these parameters, demonstrating that the last 160 ns REMD simulations nicely converged. Unless specified, all the REMD simulation results presented below are based on the last 160 ns (*t* = 200–360 ns) simulation data generated at 310 K.

### C_60_ and C_60_(OH)_8_ Inhibit the Formation of β-sheet, Especially the Long β-sheet, of hIAPP Dimer

**Figure 1:**
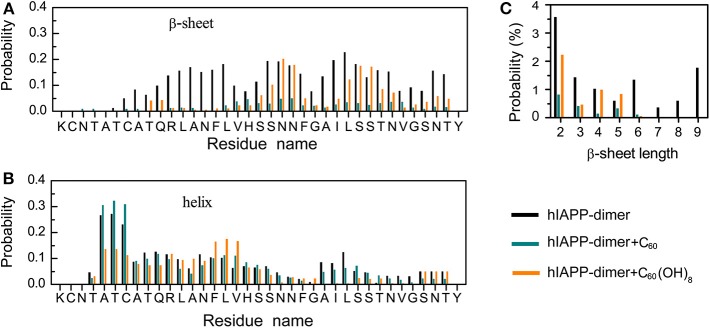
Influence of C_60_ and C_60_(OH)_8_ on the secondary structures of hIAPP dimer. Probabilities of β-sheet **(A)** and coil **(B)** as a function of amino acid residue. **(C)** The probability distribution of β-sheet length.

We first examined the percentages of different types of secondary structure formed by hIAPP dimer in each REMD system and the results were listed in Table 1. For the isolated hIAPP dimer, the probabilities of coil and β-sheet are 40.5 and 10.6%, respectively. The secondary structure propensities are in good agreement with previous circular dichroism (CD) studies (Kayed et al., 1999; Goldsbury et al., 2000) and with our recent REMD simulation results using AMBER99SB-ILDN force field (Lao et al., 2019). In comparison of the secondary structure content of isolated hIAPP dimer, the β-sheet contents of hIAPP dimer with C_60_ or C_60_(OH)_8_ are reduced from 10.6% (hIAPP-dimer system) to 1.8% (hIAPP-dimer + C_60_ system) or 4.2% (hIAPP-dimer + C_60_(OH)_8_ system) and the probability of coil increases from 40.5 to 46.7% or 48.2%. The influences of C_60_/C_60_(OH)_8_ on the probabilities of helix, turn, bend, and β-bridge structures are minor. The dominant secondary structure (β-sheet and coil) propensities of each residue of hIAPP dimer in the three systems are presented in Figures 1A,B. Figure 1A shows that residues in Q10–L16, S20–S29, and T30–T36 regions have the highest probabilities (7.0–22.7%) to form β-sheets. The three regions are reported to be the amyloidogenic regions in many experimental studies (Jaikaran et al., 2001; Nielsen et al., 2009; Bedrood et al., 2012; Zhang et al., 2013; Weirich et al., 2016). With the addition of C_60_, those β-sheet rich regions all display a reduced β-sheet probability (0.01–4.9%) (the dark cyan bars in Figure 1A). In hIAPP-dimer + C_60_(OH)_8_ system, except for polar residues N21, N22, S28, and S29, other residues including hydrophobic residues F23–L27 located in the primary amyloid core region (S20–S29) (Goldsbury et al., 2000; Brender et al., 2008; Dupuis et al., 2011) exhibit a dramatically decreased β-sheet probability (the orange bars in Figure 1A). The coil propensity of all these residues increases modestly in the presence of C_60_ or C_60_(OH)_8_. As shown in Figure 1C, nanoparticles also affect the probability distribution of β-sheet length. Upon addition of C_60_ and C_60_(OH)_8_, the probabilities of long β-sheets almost disappear. The probabilities of short β-sheets also decrease dramatically in the presence of C_60_, while their change becomes less prominent in hIAPP dimer + C_60_(OH)_8_ system. Taken together, these data demonstrate that C_60_ and C_60_(OH)_8_ substantially suppress hIAPP β-sheet formation, especially in the amyloidogenic core region, S20–S29.

**Table 1:** Secondary structure probabilities of hIAPP dimer in the absence or presence of carbon nanoparticles.

**System**	**Secondary structure content (%)**					
	**Coil**	**β-sheet**	**β-bridge**	**Bend**	**Turn**	**Helix**
hIAPP-dimer	40.5 ± 0.2	10.6 ± 0.4	2.6 ± 0.1	27.3 ± 0.4	11.5 ± 0.4	7.5 ± 0.1
hIAPP-dimer + C_60_	46.7 ± 0.2	1.8 ± 0.1	3.3 ± 0.4	28.6 ± 0.2	12.5 ± 0.05	7.0 ± 0.1
hIAPP-dimer + C_60_(OH)_8_	48.2 ± 1.4	4.2 ± 0.1	2.1 ± 0.06	29.7 ± 0.8	10.6 ± 0.3	5.2 ± 0.1

### Both C_60_ and C_60_(OH)_8_ Significantly Inhibit the Formation of β-hairpin Amyloid Precursor of hIAPP and Induce the Formation of Coil-Rich Conformations

**Figure 2:**
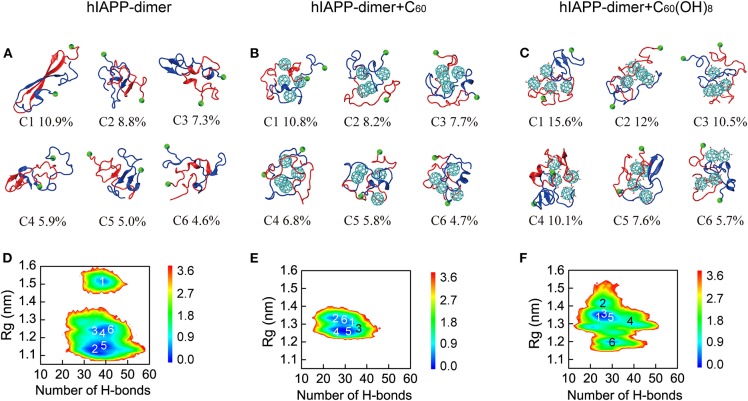
Analysis of 3D conformational properties and 2D free energy landscape of hIAPP dimer with and without C_60_/C_60_(OH)_8_. Representative conformations of the first six most-populated clusters for hIAPP dimer in hIAPP-dimer **(A)**, hIAPP-dimer + C_60_ **(B)**, and hIAPP-dimer + C_60_(OH)_8_ **(C)** systems. The corresponding population of each cluster is given below the snapshots. Free energy landscape (in kcal/mol) of hIAPP dimer as a function of the total number of H-bonds and Rg for three systems **(D–F)**. The numbers in the PMF correspond to the cluster index. The green balls refer to the C_α_ atoms of the N-terminal residue K1.

We first performed a RMSD-based cluster analysis for each REMD system at 310 K using a C_α_-root-mean-square deviation (C_α_-RMSD) cutoff of 0.35 nm to investigate the three-dimensional (3D) conformations of hIAPP dimer in the three systems. The conformations of hIAPP dimer in the three systems were separated into 85, 73, and 68 clusters, respectively. Figure 2 showed the centers of the top six most populated clusters, which represent 42.5, 44.0, and 61.5% of all conformations, respectively, for the three systems. As shown in Figure 2A, hIAPP dimer transiently adopts a three-stranded antiparallel β-sheet structure with a β-hairpin, which was considered to be hIAPP amyloidogenic precursor (Dupuis et al., 2009, 2011; Qiao et al., 2013). In hIAPP dimer + C_60_ system, this β-hairpin structure disappears and both intra- and inter-chain β-sheet content dramatically reduced, leading to collapsed disordered coil-rich conformations (Figure 2B). In hIAPP dimer + C_60_(OH)_8_ systems, inter-chain β-sheets are significantly reduced while a few intra-chain β-sheets (including short β-hairpins) still exist.

To have an overall view of the effects of C_60_ and C_60_(OH)_8_ on the whole space of conformations of hIAPP dimer, we plotted the two-dimensional (2D) free energy landscape as a function of H-bond number and Rg. It can be seen from Figure 2D that there are three minimum-energy basins of the free energy surface of isolated hIAPP dimer, located at (H-bond number, Rg) values of (40.0, 1.52 nm), (40.0, 1.23 nm), and (38.0, 1.14 nm). The first basin with the largest Rg values corresponds to the three-stranded antiparallel β-sheet structure with a β-hairpin as mentioned earlier. In the presence of C_60_ (Figure 2E), hIAPP dimer has only one narrow and deep basin located at (30.0, 1.28 nm), corresponding to collapsed disordered dimers. It is noteworthy that the basin located at (40.0, 1.52 nm) disappears, indicating that the β-hairpin amyloid precursor of hIAPP is completely suppresses in presence of C_60_. With C_60_(OH)_8_ (Figure 2F), the free energy landscape becomes shallower than that of the isolated hIAPP dimer and has a basin centered at (28.0, 1.35 nm). The decreased number of H-bond and the increased range of Rg imply a collapsed and loosely packed coil-rich hIAPP dimer.

### Both C_60_ and C_60_(OH)_8_ Weaken the Inter- and Intra-peptide Interactions of hIAPP Dimer

**Figure 3:**
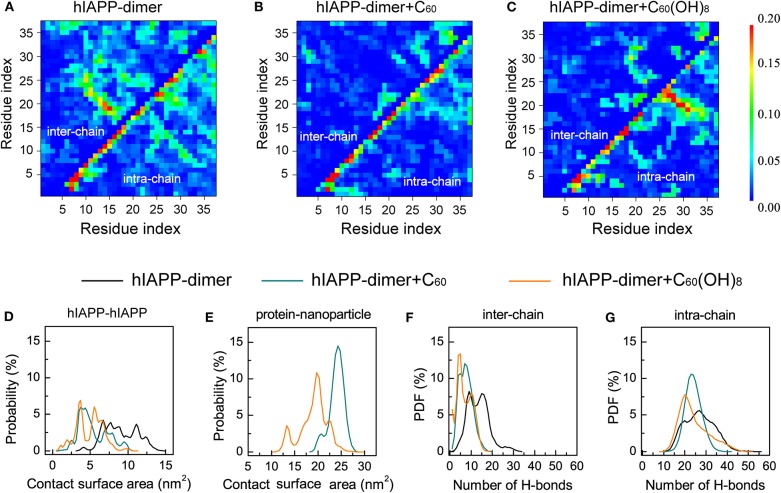
Analysis of the effects of C_60_/C_60_(OH)_8_ on the hIAPP interactions. Inter- and intra-peptide MC-MC contact probability maps for hIAPP dimer in the three different systems, hIAPP-dimer **(A)**, hIAPP-dimer + C_60_ **(B)**, and hIAPP-dimer + C_60_(OH)_8_ **(C)**. PDF of protein–protein **(D)** and protein–nanoparticle **(E)** contact surface area (CSA). PDF of inter-chain **(F)** and intra-chain **(G)** H-bond number.

To explore the effects of C_60_ and C_60_(OH)_8_ on the hIAPP interactions, we plotted the pairwise residue inter-peptide and intra-peptide MC–MC (Figures 3A–C) and SC–SC (Figure S6) contact probabilities of hIAPP dimer in the three systems. The maps of contact probability in these three systems display distinct interaction patterns, suggesting that both inter-peptide and intra-peptide interactions are remarkably altered by C_60_ and C_60_(OH)_8_. As shown in Figure 3A, without nanoparticles, residues V17–L27 and C7–V17 present the dominant probabilities of inter-chain contact. The highest probabilities of intra-chain MC–MC contact show strong anti-diagonal contacts between A8–L16 and A25–G33, suggesting the appearance of the amyloid precursor β-hairpin structure that many studies previously reported (Jiang et al., 2009; Xu et al., 2009; Qi et al., 2014; Zhao et al., 2015; Qiao et al., 2016). In the presence of C_60_, the β-hairpin pattern disappears and both the inter- and intra-chain contact probabilities are observably reduced (Figure 3B). With C_60_(OH)_8_ molecules, the inter-chain contact probabilities greatly decrease, and the amyloid precursor β-hairpin structure pattern disappears. Other short β-hairpin structures, such as that formed between S28–N31 and S19–N22, were also observed (Figure 3C). The results above demonstrate that C_60_ can markedly block both intra- and inter-peptide interactions critical for hIAPP aggregation, while C_60_(OH)_8_ dramatically alters intra-peptide interaction patterns and weakens inter-peptide interactions.

We also analyzed the contact surface area (CSA) probability between the two hIAPP monomers. without nanoparticles, the average inter-chain CSA value is 9.0 nm^2^. In the presence of C_60_/C_60_(OH)_8_ (Figure 3D), the average inter-chain CSA reduces to 5.3/4.9 nm^2^ while the CSA between hIAPP and nanoparticles is large (Figure 3E), indicating a strong binding between nanoparticles and hIAPP. We also find that hIAPP has a larger contact surface area with C_60_ than C_60_(OH)_8_, resulting in a more prominent inhibitory effect of C_60_. We further calculated the probability density function (PDF) of H-bond number formed within hIAPP dimer in the three systems. As shown in Figures 3F,G, in the presence of C_60_ or C_60_(OH)_8_, the numbers of inter-chain H-bonds are dramatically decreased as a result of the interactions between hIAPP and nanoparticles. These data suggest that the hIAPP-C_60_/C_60_(OH)_8_ interaction is stronger than hIAPP-hIAPP interaction, thus weaken hIAPP-hIAPP interactions and prevent hIAPP aggregation. Our result is consistent with recent studies showing that whether nanoparticles can inhibit or prevent peptide aggregation depends on the competition between peptide-peptide and peptide-nanoparticle interactions (Gladytz et al., 2016; Ke et al., 2019).

### C_60_ Preferentially Binds to Hydrophobic and Aromatic Residues, While C_60_(OH)_8_ Has a Relatively High Probability to Bind to Hydrophilic and Aromatic Residues

**Figure 4:**
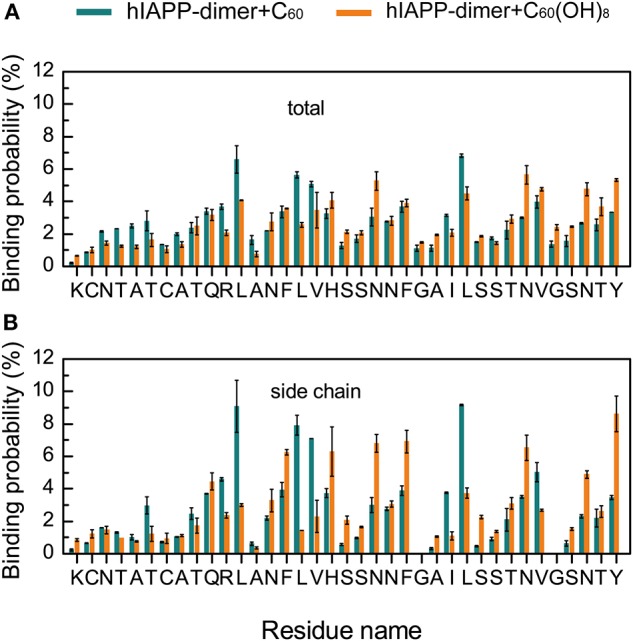
Binding probabilities of C_60_/C_60_(OH)_8_ with hIAPP dimer. Binding probabilities of C_60_/C_60_(OH)_8_ with all the atoms **(A)** and the side chain atoms **(B)** of each residue in hIAPP-dimer + C_60_ and hIAPP-dimer + C_60_(OH)_8_ systems.

To identify the most favorable binding sites of nanoparticles, we calculated the contact probabilities of C_60_/C_60_(OH)_8_ with each amino acid residue of hIAPP. Figures 4A,B shows that C_60_ nanoparticles have a relatively high probability to bind with the hydrophobic residues L12, L16, V17, L27, V32, and aromatic residues F15, F23, and Y37, reflecting that both hydrophobic and aromatic interactions play an important role in inhibiting hIAPP dimerization. It is well-known that π-π stacking of aromatic residues is crucial to the amyloid fibril formation (Azriel and Gazit, 2001; Gazit, 2002; Porat et al., 2004). It can be seen from Figures 4A,B that C_60_ and C_60_(OH)_8_ both have high propensities to interact with F15, F23, and Y37, which are the only three aromatic residues and proposed to be of great importance in hIAPP aggregation (Padrick and Miranker, 2001; Marek et al., 2007). We also find that C_60_(OH)_8_ has a relatively high probability to bind with hydrophilic residues H18, N21, N31, and N35. The hydroxylation of C_60_ makes it more hydrophilic than pristine C_60_, which weakens its interactions with hydrophobic amino acids and enhances its interactions with hydrophilic amino acids. Therefore, C_60_ and C_60_(OH)_8_ display different binding sites on hIAPP.

**Figure 5:**
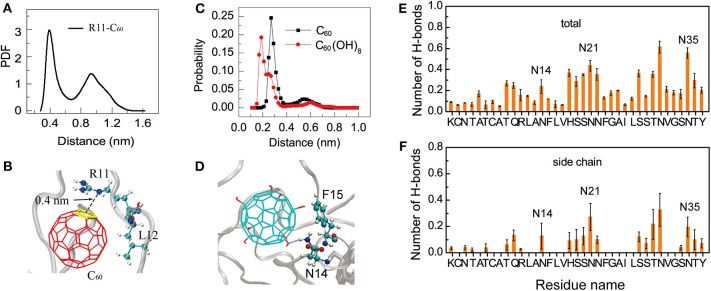
Binding mechanism of C_60_/C_60_(OH)_8_ on hIAPP dimer. **(A)** PDF of the minimum distance between NE atom of R11 and the geometry center of the C_60_ ring; **(B)** a representative snapshot showing the cation-π interaction between the atom NE in residue R11 and the ring of C_60_; **(C)** probability distribution of the minimum distance between N14/N21/N35 and C_60_/C_60_(OH)_8_. **(D)** A representative snapshot showing the collective hydrophilic and aromatic-stacking interactions between C_60_(OH)_8_ and hIAPP. **(E)** Total number of H-bonds between C_60_(OH)_8_ and each residue of hIAPP. **(F)** Number of H-bonds between C_60_(OH)_8_ and the side chain of each hIAPP residue.

Interestingly, we find that, the positively charged residues R11 in hIAPP dimer have relatively high binding probabilities with the hydrophobic C_60_. Thus, we calculated the minimum distance distribution between the atom NE of the side chain of R11 and the geometry center of each ring of C_60_. The distance distribution curve in Figure 5A shows that there exists a sharp peak centered at 0.40 nm, indicating strong cation-π interactions between R11 and C_60_ (Figure 5B). We can see from Figure 4 that C_60_ has a high contact probability with hydrophobic residue L12, this strong hydrophobic interaction might induce the cation-π interaction between R11 and C_60_.

Another interesting phenomenon is that C_60_ has stronger hydrophobicity than C_60_(OH)_8_, but C_60_(OH)_8_ exhibits much higher binding probability with the side chains of aromatic residues F15, H18, F23, and Y37 than C_60_ (Figure 4B). Meanwhile we noticed that C_60_(OH)_8_ displays high interacting probabilities with their neighboring residues N14, N21, and N35. To understand this observation at atomic level, we computed the minimum distance distribution between N14/N21/N35 and C_60_/C_60_(OH)_8_. The distance distribution curve in Figure 5C shows that the distance between C_60_(OH)_8_ and residue N14/N21/N35 is much shorter than that between C_60_ and N14/N21/N35. This result is probably attributed to the H-bond formation between the hydroxyl group of C_60_(OH)_8_ and Asn. Thus, we calculated the H-bond number between C_60_(OH)_8_ and each residue of hIAPP and found that residues N14, N21, and N35 all have high propensities to form H-bonds with C_60_(OH)_8_ (Figures 5E,F). These results suggest that the relatively high binding probability of C60(OH)_8_ with the aromatic residues F15, F23, and Y37 results from the cooperative contribution of aromatic interactions between F15, F23, Y37, and C_60_(OH)_8_, and hydrogen bonding interactions between their nearest neighboring residues N14, N21, N35, and C_60_(OH)_8_ (Figure 5D). We also find four consecutive hydrophilic residues S19, S20 N21, and N22 in the amyloid core region and four consecutive hydrophilic residues S28, S29, T30, and N31 in the C-terminal region that have high propensities to form H-bonds with C_60_(OH)_8_. It indicates that hydrogen bonding interaction between C_60_(OH)_8_ and hydrophilic residues of hIAPP also plays a role in inhibiting hIAPP aggregation.

### Both C_60_ and C_60_(OH)_8_ Weaken the Protein–Protein Interactions and Disrupt the hIAPP Protofibril

**Figure 6:**
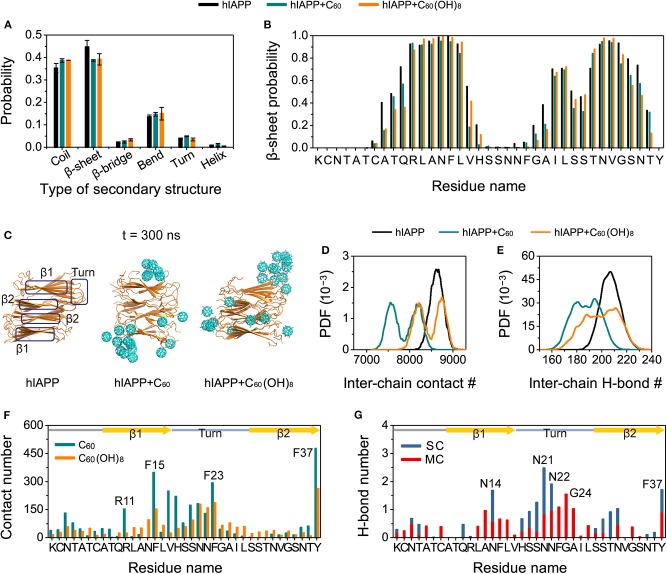
Influences of C_60_ and C_60_(OH)_8_ on the hIAPP protofibril. The average probability of each type of secondary structure **(A)**, the β-sheet probability of each amino acid of hIAPP **(B)**, the snapshots of hIAPP protofibril at *t* = 300 ns **(C)**, the PDF of the inter-chain contact number (abbreviated as **#**) **(D)** and the inter-chain H-bond number **(E)** for hIAPP-fibril, hIAPP-fibril + C_60_, and hIAPP-fibril + C_60_(OH)_8_ systems, contact number between C_60_/C_60_(OH)_8_ and each amino acid of hIAPP **(F)**, the number of H-bond formed between C_60_(OH)_8_ and the main-chain (MC) and side-chain (SC) atoms of each amino acid **(G)**. The β1, β2, and turn regions are highlighted by rectangles in **(C)** and by yellow arrows and light blue line in **(F,G)**.

We further performed MD simulations to probe into the effects of C_60_/C_60_(OH)_8_ on preformed hIAPP protofibrils. As shown in Figures 6A,B, compared to the β-sheet content in hIAPP-protofibril system (44.66%), β-sheet probabilities are reduced in both the hIAPP-protofibril + C_60_ (38.67%) and hIAPP-protofibril + C_60_(OH)_8_ (39.16%) systems, especially for the N- and C-terminal residues of the β-sheet regions (β1 and β2). It should be pointed out that the β-sheet disruption by C_60_/C_60_(OH)_8_ is less pronounced than β-sheet inhibition (β-sheet probability: 10.6% in hIAPP-dimer system, 1.8% in hIAPP-dimer + C_60_ system and 4.2% in hIAPP-dimer + C_60_(OH)_8_ system). The snapshots in Figure 6C shows that all the C_60_ and C_60_(OH)_8_ molecules bind to the surface of hIAPP protofibril. It can be seen in Figure 6F that the favorite binding sites of C_60_ and C_60_(OH)_8_ are the three aromatic amino acids, F15, F23, and Y37, revealing that the π-π staking may play a crucial role in the protein–nanoparticles interactions. F15 and F23 are located, respectively, in the C-terminal region of the β1 and the turn region of the protofibril, and Y37 is near the turn region in space. In addition, other residues with high binding probabilities are mostly located in the regions of the turn and the C-terminal of β1. These results demonstrate that both C_60_ and C_60_(OH)_8_ prefer to bind to the turn and the C-terminal of β1. The strong protein—C_60_/C_60_(OH)_8_ interactions significantly weaken and remodel the protein–protein interactions (Figures 6D,E, Figures S7A–F, S8). It is noted that C_60_(OH)_8_ displays a less prominent protofibril disruptive effect than C_60_, indicating the H-bonds (Figure 6G) formed between hydroxyl groups of C_60_(OH)_8_ and hIAPP might have limited disruptive effects on the protein–protein interactions. Taken together, both C_60_ and C_60_(OH)_8_ weaken the protein–protein interactions and disrupt the secondary structures.

## Discussion

In this study, we performed both REMD and MD simulations to study the effects of pristine and hydroxylated C_60_ on hIAPP aggregation. All-atom REMD simulations of hIAPP dimers reveal that C_60_ and C_60_(OH)_8_ can significantly suppress β-sheet formation of hIAPP. We found that, isolated hIAPP dimers adopt mostly disordered coil with a small proportion of short β-sheets. Interestingly, the previously proposed β-hairpin amyloidogenic precursor (Dupuis et al., 2009), contained in a three-stranded antiparallel β-sheet structure is also transiently populated. In the presence of C_60_ or C_60_(OH)_8_, the three-stranded antiparallel β-sheet structure with a β-hairpin completely disappears, resulting in disordered coil states. Protein-nanoparticle and protein-protein interaction analysis shows that C_60_ and C_60_(OH)_8_ both have strong binding with hIAPP and disrupt the peptide-peptide interactions responsible for hIAPP aggregation. These results indicate that both C_60_ and C_60_(OH)_8_ could slow down or hinder the aggregation of hIAPP. Further analyses reveal that the inhibition of hIAPP aggregation by C_60_ and C_60_(OH)_8_ is through different mechanism: hydrophobic and aromatic-stacking interactions for C_60_, and collective hydrogen bonding and aromatic-stacking interactions for C_60_(OH)_8_. MD simulations indicate that both C_60_ and C_60_(OH)_8_ are more likely to bind to the turn and the C-terminal of β1 via hydrophobic interactions, weaken the protein–protein interactions and disrupt the β-sheet of hIAPP protofibril. The obtained results are helpful for understanding the possible inhibitory mechanism of C_60_ and C_60_(OH)_8_ on hIAPP aggregation and provided valuable reference for the screening of potent amyloid inhibitors.

The β-sheet inhibition effect of C_60_/C_60_(OH)_8_ on amyloid proteins is sequence dependent. For example, this study together with our previous work shows that C_60_ can observably inhibit the β-sheet formation of Aβ_16−22_ (Xie et al., 2014) and hIAPP, while a recent MD study by Sun et al. reported that pristine C_60_ displays weak inhibitory impact on the aggregation of NACore of α-synuclein (Sun et al., 2019). Similarly, our REMD study demonstrates that C_60_ exhibits stronger inhibition capacity on hIAPP aggregation than C_60_(OH)_8_, whereas the work by Sun et al. shows that C_60_ has weaker inhibition ability on the aggregation of NACore of α-synuclein than C_60_(OH)_8_. It is noted that C_60_ and C_60_(OH)_8_ have poor water solubility, which limits their applications. This limitation can be overcome by increasing their extent of hydroxylation or their hydrophilicity through chemical modifications. Recently, it has been reported that graphene quantum dots and gold nanoparticles display excellent inhibition capacity against amyloidosis of hIAPP (Kim et al., 2018; Javed et al., 2019). Our results together with previous studies (Cabaleiro-Lago et al., 2012; Xie et al., 2014; Bednarikova et al., 2016; Nedumpully-Govindan et al., 2016; Kim et al., 2018) provide a better understanding of the inhibitory mechanism of nanomaterials targeting protein aggregation.

## Data Availability Statement

The datasets generated for this study are available on request to the corresponding author.

## Author Contributions

CB and GW conceived and designed the research. CB performed the simulations. CB, ZL, YC, and YT analyzed the simulation data. CB and GW wrote the paper and all authors approved the article.

### Conflict of Interest

The authors declare that the research was conducted in the absence of any commercial or financial relationships that could be construed as a potential conflict of interest.

## Supplementary Material

Additional data are provided, including construction of initial structures of hIAPP dimer, parameters for simulation data analyses, temperature list, and eight supplementary figures. These figures present the initial states of the hIAPP dimer in our REMD simulations, the convergence check for the REMD runs of the three systems, inter-/intra-chain SC-SC contact probability maps, inter-chain contact number, inter-chain H-bond number, and the differentials of the inter-chain contact maps. In the figures of the convergence check, several parameters were used, including H-bonds number, Rg, the average probability of each types of secondary structure, secondary structure propensity of each residue at 310 K. We use two time periods, 200–280 and 280–360 ns for analysis in all the REMD systems.
