# Supplementary material for: Pristine and Hydroxylated Fullerenes Prevent the Aggregation of Human Islet Amyloid Polypeptide and Display Different Inhibitory Mechanisms
Source: Front Chem. 2020 Feb 5;8:51. doi: 10.3389/fchem.2020.00051 (PMC7013002; doi:10.3389/fchem.2020.00051)
Supplement: Supplementary file 1 [file Table_1.DOCX]

Supplementary Material

**This material contains details of REMD and MD simulations,** **convergence check of REMD simulations,** **eight supplementary figures.**

1. **Construction of initial states**

The initial conformation of a hIAPP1-37 monomer with helical structure was taken from protein data bank (PDB id: 2L86). By performing a MD simulation at a high temperature of 750 K, we obtained extended coil states of hIAPP, from which 24 coil conformations were randomly selected. Every two chains were placed in cross, parallel or antiparallel orientation with a minimum distance of 1.0 nm between the two hIAPP chains (Figure S1). The exchange between two adjacent replicas was attempted every 1000 integration steps and the average acceptance ratios for each system is ~15%. Bond length of peptides and water molecules were constrained, respectively, using the LINCS [[1](#_ENREF_1)] and SETTLE [[2](#_ENREF_2)] algorithms, allowing an integration time step of 2 fs. The protein and non-protein (water and counter ion) groups were separately coupled to an external heat bath with a relaxation time of 0.1 ps using a velocity rescaling coupling method. Electrostatic interactions were treated with the particle mesh Ewald method with a real space cutoff of 1.0 nm. The van der Waals interactions were calculated using a cutoff of 1.4 nm.


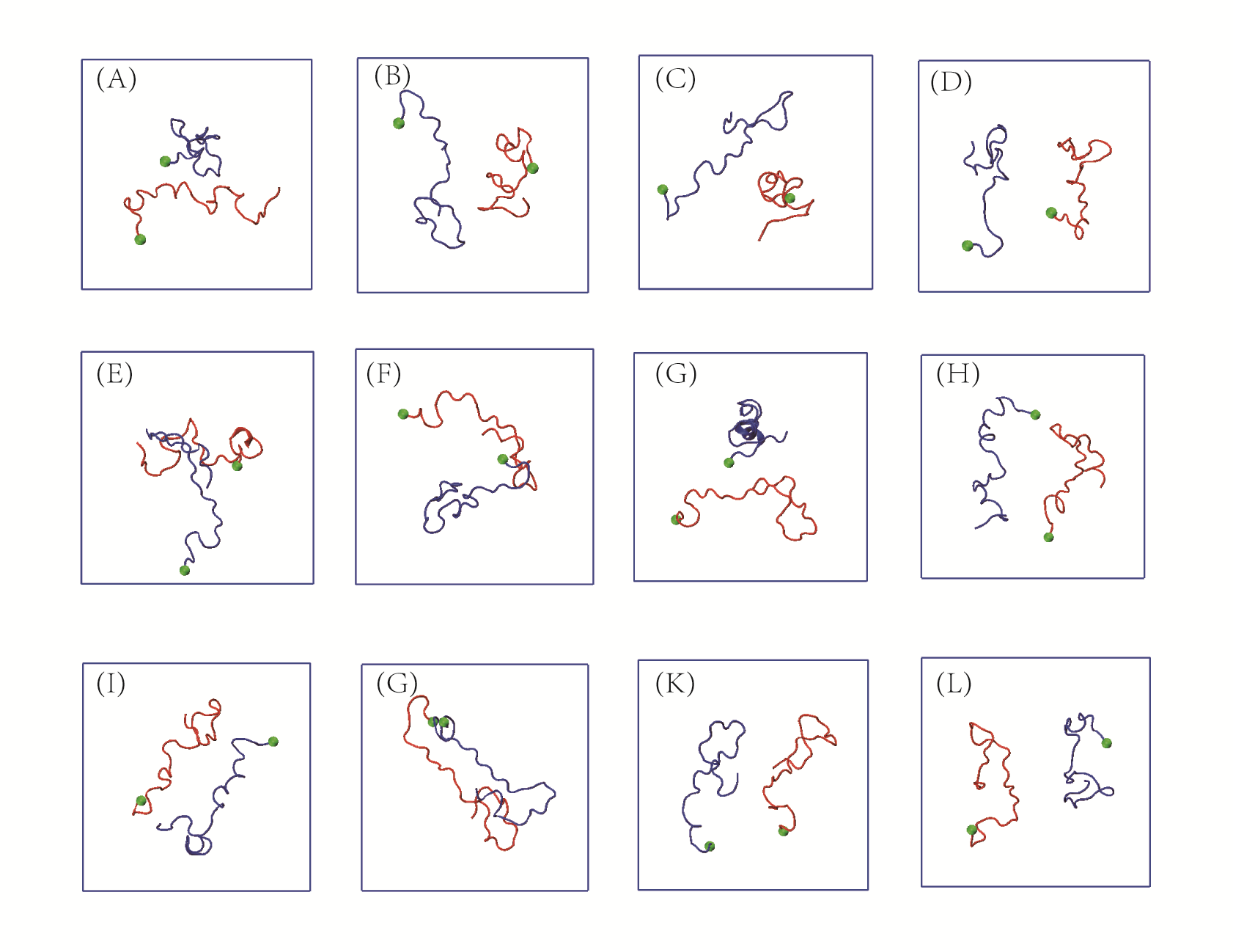


**Supplementary Figure 1.** The initial states of hIAPP dimer used for our REMD simulations. Two hIAPP chains are colored in blue and red. The green balls refer to the C_α_ atoms of the N-terminal residues.

1. **Temperature lists of REMD simulation for isolated hIAPP dimer and hIAPP dimer with nanoparticles**

The temperature list is: 306.00, 307.96, 309.92, 311.90, 313.88, 315.88, 317.89, 319.89, 321.92, 323.95, 326.00, 328.06, 330.12, 332.20, 334.29, 336.38, 338.49, 340.61, 342.74, 344.88, 347.03, 349.19, 351.36, 353.54, 355.73, 357.94, 360.16, 362.38, 364.62, 366.87, 369.13, 371.40, 373.68, 375.97, 378.28, 380.60, 382.92, 385.25, 387.60, 389.96, 392.27, 394.66, 397.06, 399.47, 401.90, 404.33, 406.78, 409.24 K.

1. **Convergence check of REMD simulations**

We checked the convergence of the simulations by comparing each of the following four parameters of hIAPP dimer within two time intervals (200-280 ns & 280-360 ns) at 310 K, including the probability density functions (PDF) of the H-bonds (Figure S2) and Rg (Figure S3), the average probability of each secondary structure (Figure S4), residue-based secondary structure propensity (Figure S5).


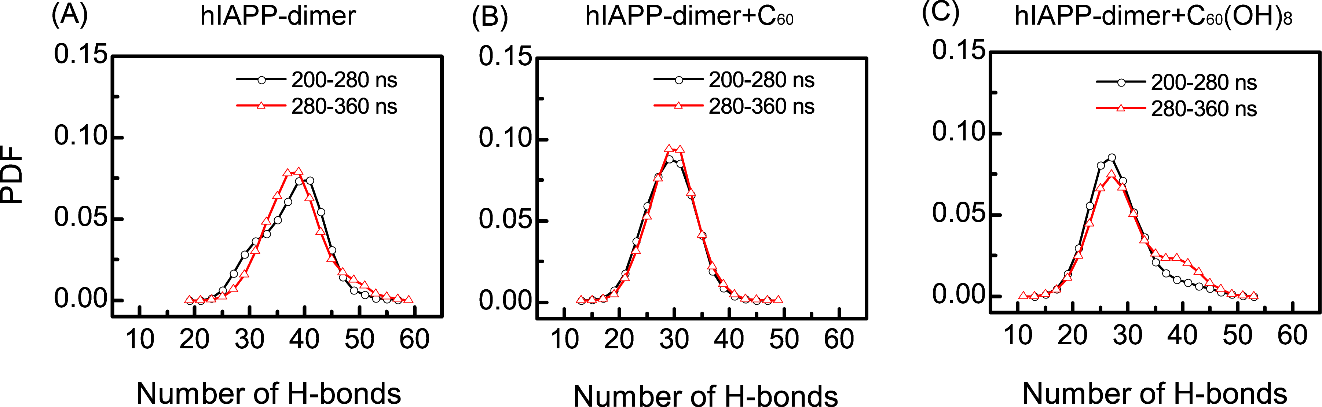


**Supplementary Figure 2.** Probability density function (PDF) of the number of hydrogen bonds (H-bonds) of hIAPP dimer in the four different system: **(A)** hIAPP-dimer; **(B)** hIAPP-dimer+C_60_; **(C)** hIAPP-dimer+C_60_(OH)_8_ within two different time intervals.


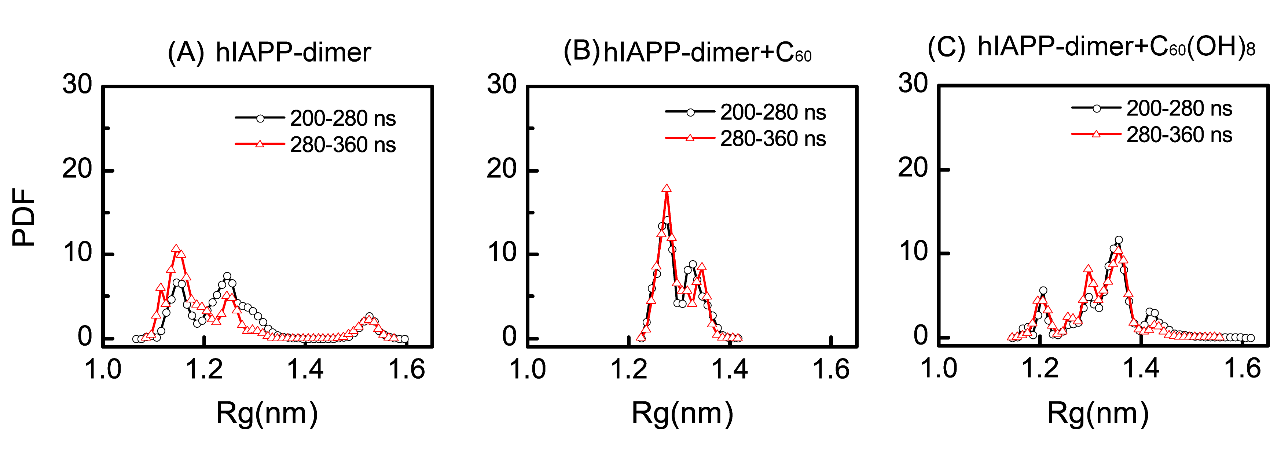


**Supplementary Figure 3.** Probability density function (PDF) of radius of gyration (Rg) of hIAPP dimer in the four different systems: **(A)** hIAPP-dimer; **(B)** hIAPP-dimer+C_60_; **(C)** hIAPP-dimer+C_60_(OH)_8_ within two different time intervals.


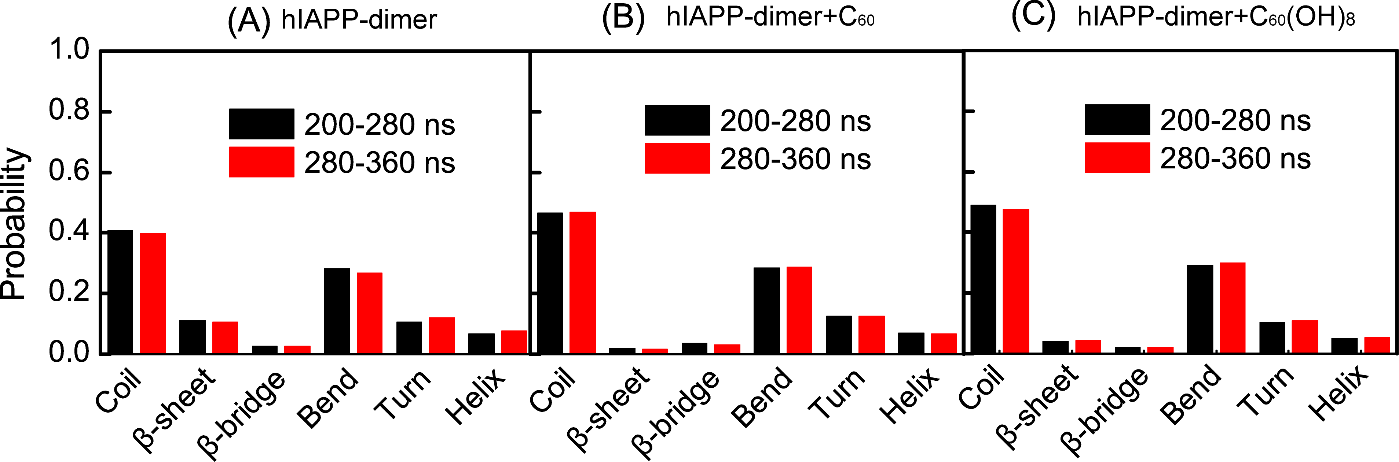


**Supplementary Figure 4.** The probability of each type of secondary structure (including coil, β-sheet, β-bridge, bend, turn and helix) averaged over all residues of hIAPP dimer in hIAPP-dimer **(A)**, hIAPP-dimer+C_60_ **(B)** and hIAPP-dimer+C_60_(OH)_8_ **(C)** systems using two different time intervals.


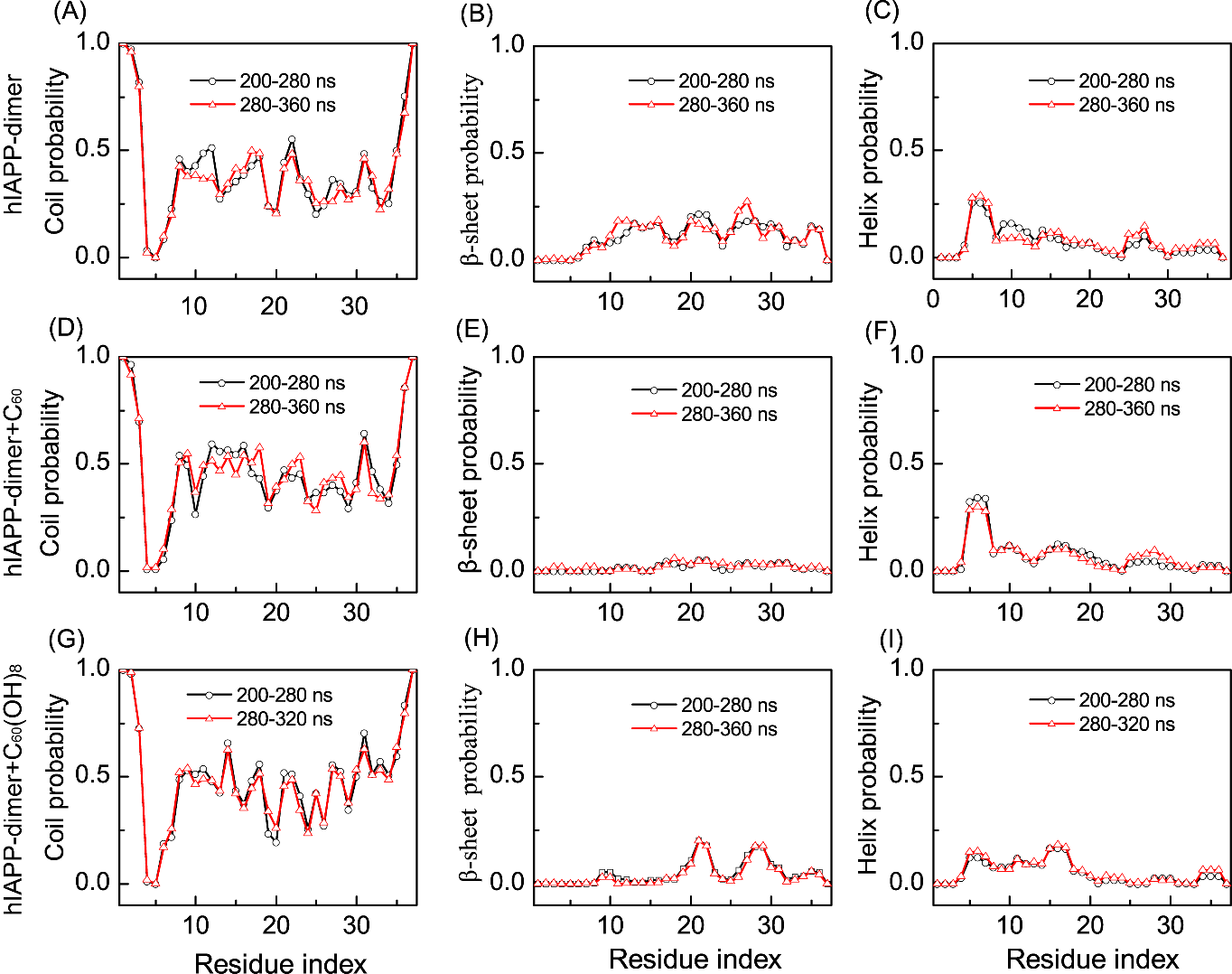


**Supplementary Figure 5.** Secondary structure propensity as a function of amino acid residue within two time intervals for the hIAPP-dimer **(A, B** and **C)**, hIAPP-dimer+C_60_ **(D, E** and **F)** and hIAPP-dimer+C_60_(OH)_8_ **(G, H** and **I)** systems.

1. **supplementary figures**


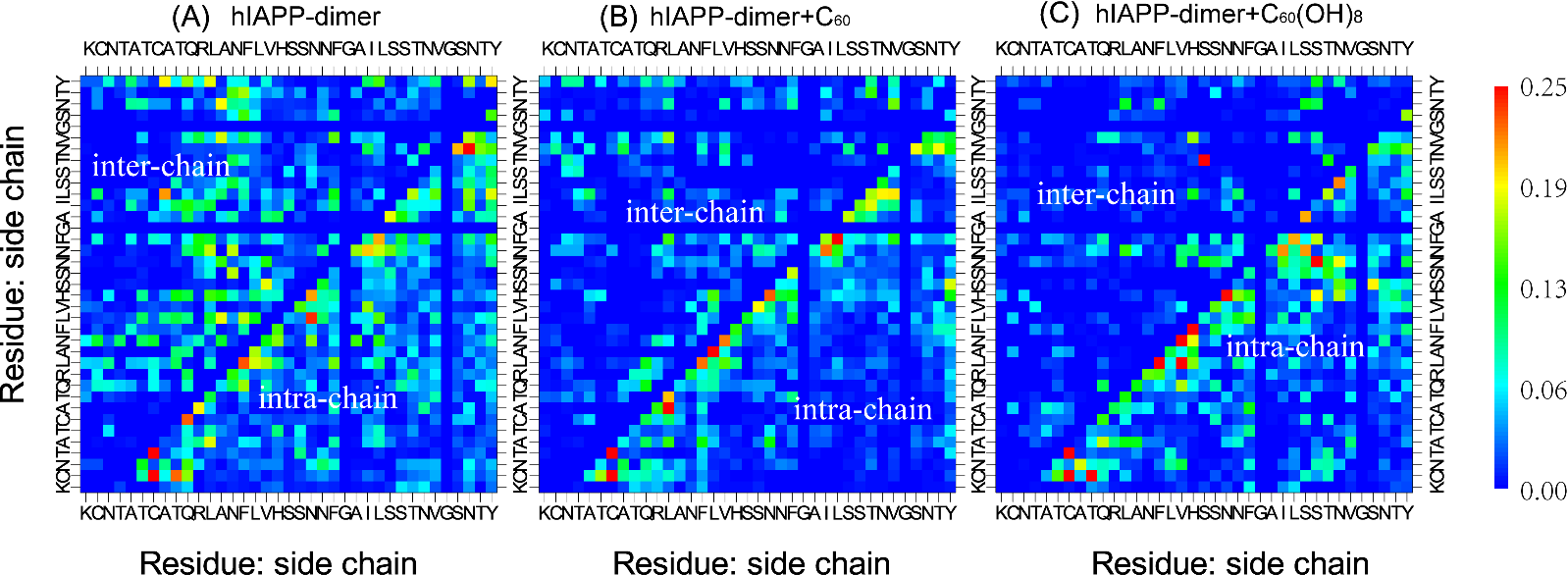


**Supplementary Figure 6.** Inter- and intra-chain pairwise residue SC-SC contact probability maps for isolated hIAPP dimer **(A)**, hIAPP dimer in the presence of C_60_ **(B)** and C_60_(OH)_8_ **(C)**.


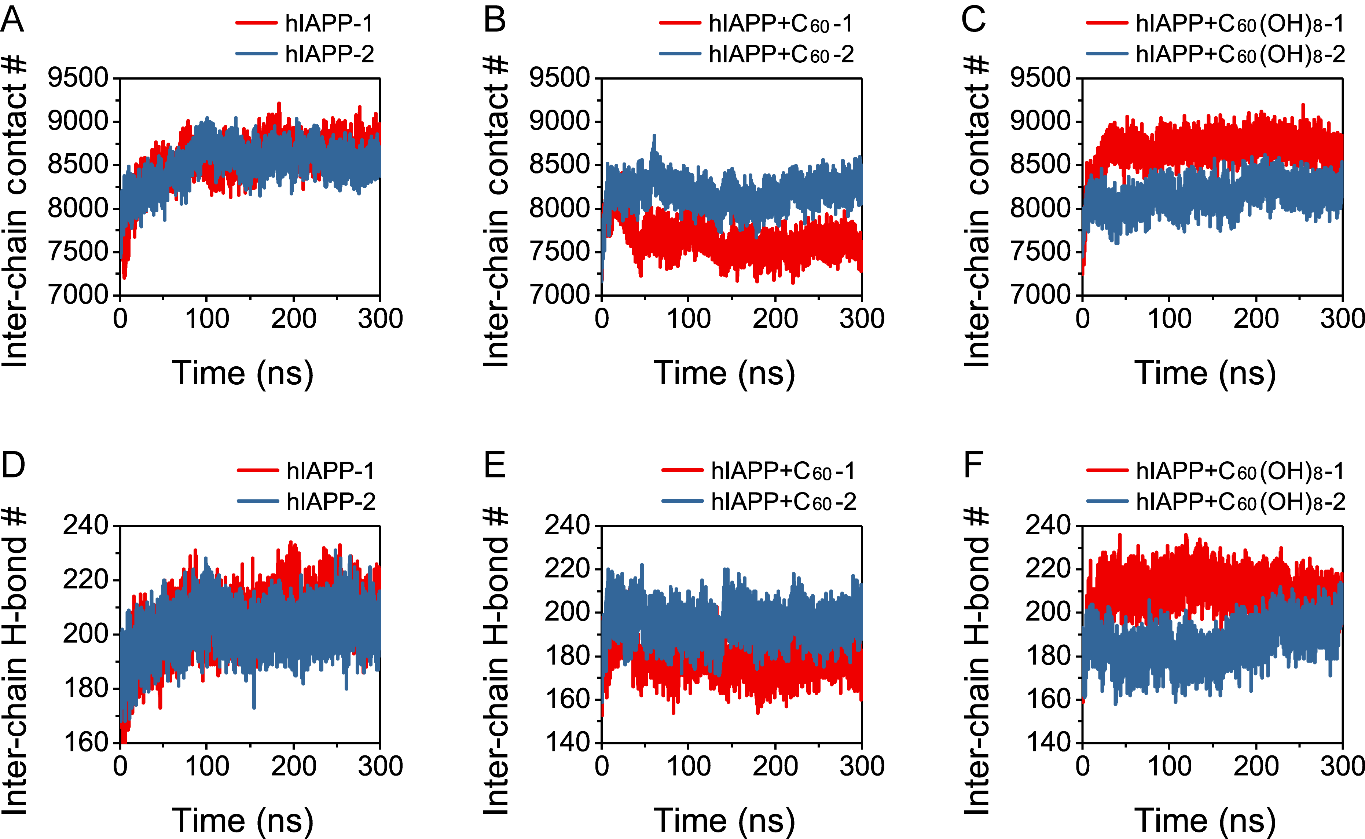


**Supplementary Figure 7.** Inter-chain contact number **(A-C)** and inter-chain H-bond number **(D-F)** of hIAPP protofibril in hIAPP-protofibril, hIAPP-protofibril+C_60_ and hIAPP-protofibril+C_60_(OH)_8_ systems.


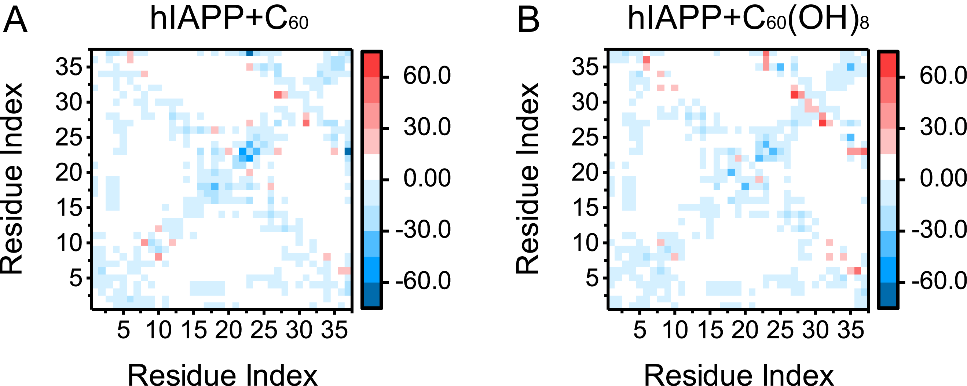


**Supplementary Figure 8.** The differentials of the inter-chain contact map between hIAPP-protofibril+C_60_ **(A)**, or hIAPP-protofibril+C_60_(OH)_8_ **(B)** and hIAPP-protofibril systems.

**Reference**

[1] Hess B, Bekker H, Berendsen HJC,Fraaije JGEM. LINCS: A linear constraint solver for molecular simulations. *J Comput Chem* (**1997)** *18*: 1463-72.

[2] Miyamoto S,Kollman PA. Settle - an Analytical Version of the Shake and Rattle Algorithm for Rigid Water Models. *J Comput Chem* (**1992)** *13*: 952-62.
